# Next-Generation Biomaterials for Load-Bearing Tissue Interfaces: Sensor-Integrated Scaffolds and Mechanoadaptive Constructs for Skeletal Regeneration

**DOI:** 10.3390/jfb16070232

**Published:** 2025-06-23

**Authors:** Rahul Kumar, Kyle Sporn, Pranay Prabhakar, Phani Paladugu, Akshay Khanna, Alex Ngo, Chirag Gowda, Ethan Waisberg, Ram Jagadeesan, Nasif Zaman, Alireza Tavakkoli

**Affiliations:** 1 Department of Biochemistry and Molecular Biology, University of Miami Miller School of Medicine, Miami, FL 33136, USA; rxk641@miami.edu (R.K.); axn668@med.miami.edu (A.N.); gowdachirag24@gmail.com (C.G.); 2 Department of Medicine, Norton College of Medicine, Upstate Medical University, Syracuse, NY 13210, USA; spornk@upstate.edu; 3 Department of Medicine, Albany Medical College, Albany, NY 12208, USA; prabhap@amc.edu; 4 Brigham and Women’s Hospital, Harvard Medical School, Boston, MA 02115, USA; phani.paladugu@students.jefferson.edu; 5 Sidney Kimmel Medical College, Thomas Jefferson University, Philadelphia, PA 19107, USA; aya156@students.jefferson.edu; 6 Department of Clinical Neurosciences, University of Cambridge, Cambridge CB2 1TN, UK; 7 Whiting School of Engineering, Johns Hopkins University, Baltimore, MD 21218, USA; ramjagad@cisco.com; 8 Smith-Kettlewell Eye Research Institute, San Francisco, CA 94115, USA; zaman@nevada.unr.edu; 9 Department of Computer Science, University of Nevada, Reno, NV 89557, USA; tavakkol@unr.edu

**Keywords:** mechanoadaptation, osteoinductive biomaterials, sensor-integrated scaffolds, load-bearing interfaces, skeletal regeneration, orthopedic tissue engineering

## Abstract

Advancements in load-bearing tissue repair increasingly demand biomaterials that not only support structural integrity but also interact dynamically with the physiological environment. This review examines the latest progress in smart biomaterials designed for skeletal reconstruction, with emphasis on mechanoresponsive scaffolds, bioactive composites, and integrated microsensors for real-time monitoring. We explore material formulations that enhance osseointegration, resist micromotion-induced loosening, and modulate inflammatory responses at the bone–implant interface. Additionally, we assess novel fabrication methods—such as additive manufacturing and gradient-based material deposition—for tailoring stiffness, porosity, and degradation profiles to match host biomechanics. Special attention is given to sensor-augmented platforms capable of detecting mechanical strain, biofilm formation, and early-stage implant failure. Together, these technologies promise a new class of bioresponsive, diagnostic-capable constructs that extend beyond static support to become active agents in regenerative healing and post-operative monitoring. This multidisciplinary review integrates insights from materials science, mechanobiology, and device engineering to inform the future of implantable systems in skeletal tissue repair.

## 1. Introduction

The evolution of skeletal regeneration strategies has progressed from passive, static structural support systems toward dynamic, bioresponsive platforms capable of interfacing intelligently with their physiological environment [1]. Increasingly, sophisticated biomaterials are being integrated into scaffold designs to not only replicate the structural integrity of native bone but also to approximate its adaptive behavior under variable mechanical loading conditions [2,3]. These advanced scaffolds are engineered to actively engage with surrounding tissues, detect and respond to biomechanical cues in situ, and, in many cases, deliver diagnostic insights through embedded sensing technologies [4].

This narrative review aims to provide a comprehensive overview of recent advancements in biomaterials for skeletal regeneration, with a focus on sensor-integrated scaffolds and mechanoadaptive constructs. Unlike a systematic review, which adheres to a predefined protocol, our approach involves a selective synthesis of key studies and emerging trends identified through a broad literature search in PubMed, Scopus, and Web of Science, targeting publications from the last decade (2013–2023). We prioritized works that highlight innovative material designs and their clinical relevance, particularly in addressing challenges such as poor osseointegration and implant failure in load-bearing applications. The relevance of this review lies in its integration of cutting-edge developments across materials science, mechanobiology, and device engineering, offering a forward-looking perspective on the future of regenerative implants.

In this review, recent innovations are examined across key domains—including mechanoresponsive scaffolds that modulate their properties in response to external forces, bioactive composite materials that synergistically support osteointegration and tissue remodeling, and multi-functional constructs equipped with embedded microsensors. Together, these next-generation platforms address longstanding challenges in the reconstruction and repair of load-bearing tissues, offering improved outcomes through enhanced biological integration, real-time monitoring, and adaptive mechanical performance [5,6].

## 2. Advanced Fabrication Techniques for Load-Bearing Scaffolds

### 2.1. Gradient Scaffold Fabrication

Biomimetic gradient scaffolds are a complex family of regenerative structures designed to reproduce the hierarchical organization and compositional continuity inherent in natural musculoskeletal tissue interfaces [7]. Similar features that replicate this transitional architecture are seen across osteotendinous, osteochondral, and corticocensellous junctions [8,9,10], and these scaffolds include regionally defined gradients in material composition, porosity, elastic modulus, and biochemical signaling. These features enhance load transfer accuracy under complex physiological loads by reducing stress concentrations at material discontinuities, hence decreasing the likelihood of mechanical failure and delamination [11]. Their graded microenvironments concurrently regulate stem cell fate decisions and extracellular matrix (ECM) deposition by spatiotemporally defined signals, hence facilitating the formation of physically anisotropic, functionally connected tissue compartments [12,13]. Currently, layer-by-layer additive manufacturing, particularly extrusion-based techniques with programmed mixing, enables the continuous deposition of hydroxyapatite–polymer gradients, therefore tailoring mineral density profiles to emulate natural enthesis transitions [14,15]. Common polymeric materials employed in these scaffolds include polycaprolactone (PCL) and poly(lactic-co-glycolic acid) (PLGA), chosen for their biodegradability and mechanical tunability. PCL offers a slow degradation profile and high toughness, ideal for sustained structural support, while PLGA allows for faster degradation and is often blended with hydroxyapatite to enhance osteoconductivity. Through integrin-mediated signaling and mechanical transduction, these compositional gradients generate localized changes in stiffness, degradation kinetics, and cellular adhesion ligand density, hence driving lineage-specific differentiation [16]. From osteogenic (10–20 GPa) to fibrocartilaginous (0.1–1 MPa), elastic modulus gradients spanning orders of magnitude produce strain differentials under cyclic loading that amplify spatially resolved mechanosensitive gene expression—a phenomenon now quantifiable using nanoindentation mapping and digital volume correlation [17,18]. Concurrently, architected porosity gradients, ranging from 90% open volume in infiltration zones to 30% in load-transmission zones, change permeability, fluid shear stress, and effective stiffness, thereby directly impacting neovascularization kinetics and cell dispersion uniformity [19]. These porosity designs are achieved by real-time tuned production techniques using variable nozzle actuation and deposition speed without sacrificing mechanical stability or interconnectivity [20]. With Raman mapping and energy-dispersive X-ray spectroscopy now providing micron-level compositional verification, mineral gradients—built through spatially controlled deposition of calcium phosphate nanoparticles—allow zone-specific osteoconductivity and differential ECM mineralization [21,22]. Through controlled-release kinetics from biodegradable microspheres, orthogonal growth factor gradients—such as BMP-2, VEGF, and PDGF—are incorporated by microfluidic patterning or inkjet deposition, thus establishing temporally evolving biochemical gradients that synchronize with healing cascades [23,24]. These gradients, taken together, provide a new paradigm in regenerative scaffold engineering—constructs not just passive templates but dynamic, spatially intelligent systems able to coordinate complicated, multi-tissue morphogenesis under biomechanically demanding conditions [25].

Using plasma intensity gradation to induce wettability transitions that guide cell migration and cytoskeletal organization across defined axes, gradient scaffold technologies leverage spatially modulated surface properties—such as wettability, charge density, and nanoscale topography—to precisely direct protein adsorption, cellular adhesion, and matrix deposition [26,27]. Now measurable by atomic force microscopy and contact angle mapping, these surface-level gradients combine with bulk material gradients to create hierarchical, multiscale cueing systems that affect cell activity by concurrent physicochemical pathways [28]. Manufacturing these gradients, however, presents novel challenges, including preserving continuous transitions, spatial fidelity, and inter-batch reproducibility. However, these issues can be mitigated by closed-loop feedback systems and real-time monitoring technologies, thereby allowing high-resolution deposition and compositional modulation [29,30]. New testing systems have been developed to evaluate the integrity and operation of such structures by quantifying gradient steepness, spatial continuity, and mechanical heterogeneity, therefore aiding both quality assurance and regulatory compliance [31]. Functional characterization under physiologically relevant loads—such as compression-tension testing at osteotendinous interfaces and shear assessments of layered composites—reveals application-specific mechanical behaviors and failure modes that standard assays fail to capture, providing critical data to refine iterative design processes [32,33]. Clinically, mineral and porosity gradient scaffolds have demonstrated superior tissue integration and mechanical resilience in complex reconstructions, namely bone–tendon and cortical–cancellous interfaces, outperforming homogeneous constructs by promoting spatially uniform tissue ingrowth and minimizing delamination risks [34,35]. The accumulating clinical and biomechanical validation of these gradient systems underscores their transformative potential in precision-guided tissue engineering and complex anatomical interface reconstruction [36].

### 2.2. Hybrid Material Systems

**Figure 1:**
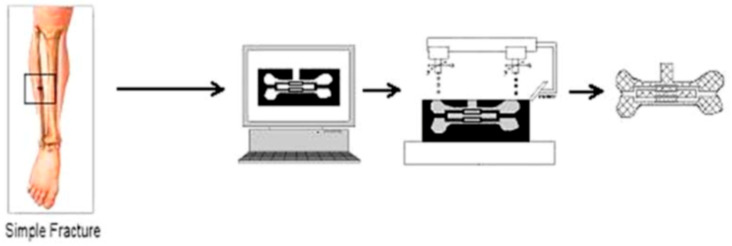
Use of solid freeform fabrication in the design of composite scaffolds. This file is licensed under the Creative Commons Attribution-Share Alike 4.0 International license with permission from Wikimedia Commons [40].

Composite scaffolds leveraging combinations of ceramics, polymers, metals, and biologically derived components have emerged as a potent approach to engineering multifunctional materials capable of addressing the complex mechanical, structural, and biological demands of load-bearing tissue interfaces [37,38]. While metal–polymer composites increase mechanical stability without undue stiffness, hence reducing stress shielding, ceramic–polymer systems blend the osteoconductivity of calcium phosphates with the elasticity and biodegradability of polymers [39]. Additives such as hydroxyapatite and bioactive glass are frequently incorporated into these matrices. Hydroxyapatite enhances osteoconductivity and mimics the mineral component of bone, while bioactive glass releases ions (e.g., calcium, phosphate, silicon) that stimulate osteogenesis and angiogenesis (Figure 1) [40].

With low loading fractions, nanocomposite formulations, including carbon nanotubes, graphene, or nanofibers, show substantial increases in mechanical performance, electrical conductivity, and bioactivity [41,42]. By producing a large interfacial area with the matrix, these nanoscale reinforcements improve load transmission and introduce multifunctionality [43]. Through obtaining high tensile strength and high hydration, hydrogel-based fiber-reinforced composites further extend this adaptability, imitating the gradient characteristics of osteotendinous and osteochondral junctions, thus supporting both mechanical demands and cellular survival [44]. Microfluidic approaches and additive manufacturing now allow exact spatial placement of these reinforcements to reproduce anisotropic tissue characteristics [45].

Moreover, these advanced composite systems exhibit dynamic or bioresponsive behavior. Embedded shape-memory materials inside scaffolds allow conformational changes in situ, therefore improving implant fixation and producing mechanotransductive impulses to promote tissue regeneration [46,47]. To stimulate osteogenesis, angiogenesis, and antibacterial activity, bioactive glass-polymer composites similarly release therapeutic ions, including calcium, phosphate, silicon, and strontium in spatially regulated gradients [48]. Natural–synthetic hybrids—such as collagen–polycaprolactone systems—combine mechanical dependability and tunability with biological recognition and degradability, thereby producing unified scaffolds with emerging biological and mechanical capability [49]. Stimulus-responsive composites comprising pH-sensitive, enzyme-degradable, or temperature-sensitive domains react adaptably to the healing environment, coordinating breakdown kinetics, mechanical transitions, or bioactive release with biological signals [50,51]. These adaptive features indicate a change toward scaffolds that not only facilitate passive regeneration but also actively contribute to its advancement [52]. It is important to note that the success of such composite systems depends critically on interfacial engineering. Bonding between several material phases determines mechanical stability, fatigue resistance, and integration integrity [53]. Related methods include surface functionalization, silane coupling, and interpenetrating phase networks, which can improve chemical and mechanical compatibility, hence reducing interfacial failure under stress [54,55].

**Figure 2:**
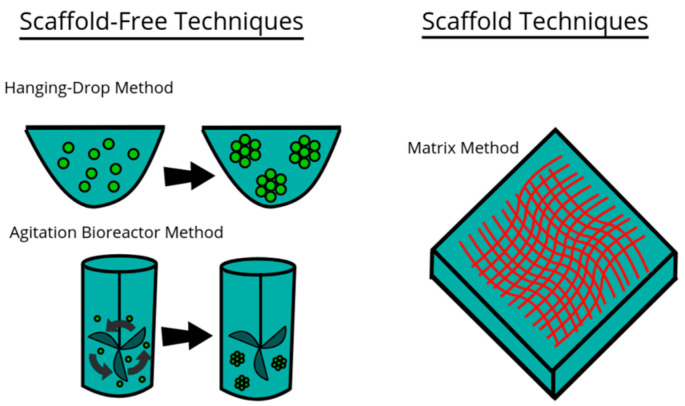
Model of three examples of techniques used for culturing cells in 3D environments. This file is licensed under the Creative Commons Attribution-Share Alike 4.0 International license with permission from Wikimedia Commons [59].

Advances in powder-based manufacturing (e.g., selective laser melting) and surface activation technologies [56] additionally enable metal–ceramic composites—such as titanium-reinforced calcium phosphate scaffolds—to achieve compressive strengths exceeding 150 MPa and elastic moduli within the physiological range of cortical bone [57]. Biphasic and triphasic ceramic–ceramic systems further enable spatial and temporal control of degradation, tailoring ion release and scaffold resorption to match site-specific healing kinetics. From ongoing studies, it is evident that dynamic interfaces able to adjust during healing will define the next frontier in regenerative biomaterials, particularly as composite scaffold design becomes more multi-material and multi-functional (Figure 2) [58].

## 3. Sensor-Integrated Scaffolds for Real-Time Monitoring

### 3.1. Microsensor Networks for Strain Detection

A major breakthrough in regenerative implant design is embedded microsensor networks within three-dimensional scaffold structures (Table 1). These networks provide continuous, high-resolution monitoring of mechanical microenvironments throughout the osseointegration and remodeling stages [60,61]. Early identification of subclinical events, including micromotion-induced loosening, stress shielding, or impaired load transmission—pathophysiological precursors to implant failure traditionally undetectable by static radiography or delayed symptomology—these sensorized constructions detect spatially resolved strain distribution [62]. High gauge factors (>50) piezoresistive sensors made of carbon-based nanocomposites and conductive polymers combine easily into scaffold struts while maintaining load-bearing capacity and biofunctionality [63]. Advanced designs broadcast continuous data by wireless telemetry to external receivers [64], hence enhancing strain sensitivity to detect micromotions 20 μm. Because of their micro-scale form factor and high biocompatibility, complementary optical sensing platforms—including fiber Bragg gratings and Fabry–Perot interferometric systems—achieve submicron strain resolution (<0.1 μm), immune to electromagnetic noise, and compatible with dense implant geometries [65,66]. Using tuned conductivity for ultra-sensitive detection, graphene-based sensors fit to anatomically complicated topographies [67] and provide multifarious monitoring—strain, temperature, pH—within atomic-scale thickness. Dense sensor arrays implanted along anisotropic scaffold axes are made possible by fabrication using photolithographic thin-film patterning and microelectromechanical systems (MEMS) technology, therefore permitting spatiotemporal tracking of mechanical cues essential to mechanotransduction-driven differentiation [68]. Non-fouling surface treatments help to reduce inflammatory cascades by means of encapsulation techniques using parylene-C and silicone elastometers [69]. By means of finite element modeling, mechanical congruence between sensor materials and scaffold matrices is computationally adjusted to prevent local stress risers and, hence, preserve build integrity during cyclic loading [70]. Each adjusted to scaffold-specific energy needs, biomechanically active piezoelectric harvesters, microscale supercapacitors, and long-life biocompatible microbatteries address energy autonomy [71,72]. Clinically, in high-risk reconstructive settings—monitoring fusion kinetics in spinal arthrodesis, identifying early aseptic loosening in arthroplasty components, and estimating strain development across fracture nonunions—these smart scaffolds provide hitherto unheard-of diagnostic resolution [73]. Ongoing validation studies are now establishing quantitative thresholds for clinical decision-making, positioning sensor-integrated scaffolds as next-generation therapeutic-diagnostic (“theranostic”) platforms within precision musculoskeletal repair [74].

### 3.2. Biofilm Detection and Infection Control

Primarily due to biofilm development, which generates antibiotic-resistant microbial reservoirs that escape immune clearance [75], bacterial infection remains a significant problem in skeletal restoration. Next-generation scaffolds now have improved biosensing capabilities and are able to detect early bacterial colonization—well before full biofilms develop (Table 1) [76]. With detection limits below 10^3^ CFU/cm^2^, techniques such as electrochemical impedance spectroscopy (EIS) identify impedance changes on scaffold surfaces generated by microbial adherence and EPS secretion, therefore enabling label-free, real-time detection [77]. Targeting quorum sensing molecules (e.g., AHLs, AIPs), complementary methods use synthetic receptors to identify bacterial communication signals suggestive of early-stage infection [78]. Additionally, pH-sensitive probes detecting localized acidification from bacterial metabolism, temperature sensors tracking inflammation-induced heat elevations, and SERS systems offering species-specific bacterial identification via enhanced molecular fingerprinting [79,80] are analogous sensing modalities. As scattered arrays over scaffold surfaces, these sensors are increasingly combined to provide high-resolution spatial maps of colonization and infection progression [81].

Responsive scaffolds combine on-demand antimicrobial release with sensing systems to expand this capability. Sensor outputs activate smart hydrogels containing antibiotics, antimicrobial peptides, or ions to release localized therapies at the initial infection location, therefore limiting systemic exposure and the danger of resistance [82,83]. While dynamically sensitive surfaces activate antimicrobial defenses upon pathogen presence [84], these scaffolds commonly integrate passive antifouling strategies—such as SLIps, zwitterionic coatings, or bactericidal nanotopographies—to avoid initial adherence. Early infection may be distinguished from benign physiological changes by very precise interpretation of complicated biological data using advanced signal processing incorporating machine learning algorithms based on multi-modal sensor outputs [85]. These systems synthesize data across modalities—impedance, pH, thermal, and spectroscopic signatures—building strong diagnostic profiles [86]. Encapsulation methods extending sensor lifetime in vivo, wireless telemetry for continuous data transmission, and integration with current monitoring systems support clinical translation, thereby indicating a major step toward real-world deployment of autonomous, infection-resistant orthopedic implants [87].

### 3.3. pH and Metabolite Tracking

With criteria like pH, oxygen tension, and metabolite concentrations directly regulating cellular activity and tissue formation, the local biochemical environment within healing tissues greatly determines regeneration outcomes [88]. Next-generation scaffolds provide hitherto unheard-of insight into the biochemical side of the healing process, particularly by including advanced monitoring capabilities that define this environment in real time (Table 1) [89]. Unfortunately, traditional evaluation techniques, including systematic blood measures, miss the localized circumstances inside healing tissues [90]. Through high spatial and temporal resolution, integrated microsensors identify these local characteristics, thereby enabling thorough characterization of the biochemical environment across the scaffold volume [88]. This monitoring capacity converts passive scaffolds into active sensing platforms that continually assess their own internal conditions, therefore offering important data for clinical uses as well as for research [91].

In scaffold contexts, pH monitoring offers important data about cellular metabolism, tissue perfusion, and inflammatory processes [92]. Under hypoxic circumstances, optical sensors using pH-sensitive fluorophores detect local acidification resulting from cellular glycolysis; contemporary systems achieve pH resolution below 0.05 units [93]. Miniaturized ion-selective electrodes and other electrochemical techniques provide complementing capabilities with great long-term stability, allowing continuous monitoring throughout the healing process [94]. The relationship between pH patterns and healing development creates typical ranges that separate effective regeneration from pathological processes, therefore defining unambiguous thresholds for intervention when unfavorable circumstances arise [95]. By means of spatial mapping of pH across scaffold volumes, regional differences that can point to insufficient vascularization or localized inflammation can be found, therefore driving focused therapies to address these particular issues [96].

In large tissue constructions, where inadequate vascularization generally restricts oxygen supply to cells in core areas [97], oxygen tension monitoring helps mitigate hypoxia. With oxygen-sensitive phosphors buried in scaffold materials generating scattered sensing networks over the build volume, phosphorescence quenching by oxygen offers a strong optical sensing mechanism [98]. Non-invasive optical detection allows real-time analysis of oxygen distribution patterns [99] and continuous monitoring free from disturbance of the healing environment. The relationship between oxygen levels and cellular viability sets minimal thresholds needed for effective tissue regeneration, which guides vascularization techniques to provide sufficient oxygen supply across large scaffolds [100]. Recent advances, such as oxygen-generating materials that activate in hypoxic circumstances, produce responsive systems that automatically solve growing oxygen deficits [101].

With their respective concentrations suggesting main energy pathways and metabolic stress levels, glucose and lactate monitoring provide information on cellular metabolic activity [102]. Enzymatic sensors using glucose oxidase or lactate oxidase generate electrical impulses proportional to local concentrations of these crucial metabolites, hence enabling continuous monitoring capabilities [103]. Elevated lactate levels signify oxygen limitations that might threaten long-term viability, whereas the ratio of glucose consumption to lactate production indicates whether cells mostly use aerobic or anaerobic metabolism [104]. The correlation between these metabolic patterns and efficient repair establishes distinctive reference profiles for normal regeneration, facilitating the early identification of metabolic anomalies indicative of potential issues [105]. Next-generation techniques integrate many enzyme systems that simultaneously assess diverse metabolites to provide complete metabolic profiles, therefore more accurately characterizing the cellular environment [106].

The identification of inflammatory markers offers essential insights into the transition from acute to regenerative inflammation, a shift crucial for successful repair [107]. Miniature immunosensors use antibody-based identification to detect specific cytokines, such as TNF-α, IL-1β, and IL-6, therefore accurately characterizing the local inflammatory environment [108]. Complementary techniques ascertain enzyme activity, including matrix metalloproteinases (MMPs), indicating ongoing tissue remodeling or potential degradation [109]. The temporal patterns of numerous inflammatory markers enable the early diagnosis of deficient inflammatory responses that may affect outcomes, producing distinct profiles associated with successful regeneration [110]. This monitoring capacity largely challenges the conventional wisdom about inflammation from a binary perspective to a dynamic process needing certain transitions for optimal healing [111].

Active ion concentration monitoring can also help researchers understand mineralization, cellular signaling, and scaffold degradation [112]. Ion-selective electrodes and optically active ionophores can help detect certain ions like calcium, phosphate, and magnesium [113]. Similarly, potassium and sodium levels define cellular health and membrane integrity; aberrant ratios, therefore, can point to possible cellular harm [114]. Successful mineralization and these ionic profiles provide reference patterns that separate normal growth from pathological calcification or inadequate mineral deposition [115]. For degradable scaffolds with calcium phosphate components, where ion release from the scaffold immediately affects the surrounding environment and cellular responses [116], this monitoring capacity offers notable value. In addition to direct sensing techniques, integrating microfluidics with sensor systems facilitates sample collection, reagent administration, and fluid manipulation within scaffolds [117]. Small channels (50–200 μm in diameter) can be embedded within scaffold materials and help create fluid networks that connect sensor regions, thereby facilitating the sequential analysis of multiple parameters from collected interstitial fluid samples [118]. These technologies address the issue of limited sample sizes through highly effective microanalytical methods that optimize information extraction from minimal sample quantities [119]. Active microfluidic components, including valves and pumps, provide precise control over fluid flow, thereby creating robust analytical platforms within the scaffold structure [120]. Additionally, next-generation technologies offer adaptive systems that enhance monitoring operations through the incorporation of stimuli-responsive materials, which autonomously adjust fluidic pathways depending on sensed conditions [121].

Hydrogel-based sensing devices exhibit a unique combination of widespread biocompatibility and versatile sensing capabilities [122]. By including small-molecule receptors, antibodies, or enzymes in these hydrophilic polymer networks, analyte interaction produces observable signals [123]. While preserving functioning under physiological settings, these materials’ high water content and soft mechanical characteristics help reduce foreign body responses [124]. Analyte diffusion across the hydrogel matrix allows continuous monitoring free from fluidic components, hence enabling incorporation into intricate scaffold designs [125]. Recent developments combine many sensing capabilities inside single hydrogel components to provide different monitoring systems that concurrently characterize several parameters using orthogonal detection techniques [126].

In certain settings, colorimetric and fluorescent markers can provide visually detectable signals that allow non-invasive monitoring of surface tissues [127]. Phenol red and bromothymol blue, for instance, are pH-sensitive dyes that show color changes in response to local pH circumstances [128]. Thus, they can be used as clear markers of acidification to correspond with inflammation or hypoxia. With intensity giving a quantitative indication of oxygen availability, oxygen-sensitive phosphors can produce differing light intensities that correspond with local oxygen content [129]. Including these markers in transparent or translucent scaffold materials can enable visual monitoring through minimally invasive optical access ports or, for superficial uses, maybe visible through unbroken skin [130]. This approach improves monitoring for particular uses, hence lowering reliance on electronic components and power sources that prevent long-term implantation [131].

### 3.4. Antibacterial Nanostructures

Due to the rapid development of biofilms—structured microbial communities encased in extracellular polymeric matrices with greatly enhanced tolerance to antibiotics and immune-mediated clearance [132], bacterial infection remains one of the most challenging complications in clinical medicine. Resistance development and poor tissue penetration progressively hamper conventional pharmacologic treatments. Consequently, a shift in paradigm towards structurally encoded, non-leaching antimicrobial modalities is necessary [133]. While maintaining host osteoprogenitor and endothelial cell compatibility, next-generation scaffolds now incorporate nanoscale surface characteristics and responsive materials that demonstrate broad-spectrum antibacterial action via mechano-physical, ion-mediated, and oxidative processes [134]. Using biomechanical weaknesses in prokaryotic envelopes, high-aspect-ratio nanostructures such as ZnO nanowires (1–5 μm length) and nanopillars (200–300 nm tall, ~100 nm spacing) physically disrupt bacterial membranes upon contact, simultaneously avoiding negative responses in mammalian cells [135]. At the same time, immobilization techniques (e.g., polydopamine anchoring, LbL assembly) prevent cytotoxic leaching and extend functional lifespan; concurrently, ion-releasing nanomaterials such as copper oxide nanostructures foster sustained antimicrobial zones through controlled ion dissolution and reactive oxygen species (ROS). Silver (AgNPs, 10–50 nm).

Complementing these direct-kill strategies are advanced antifouling and stimuli-responsive systems that prevent bacterial adhesion or otherwise activate only under pathogenic conditions [136]. Hydrophilic polymer brushes (e.g., PEG, zwitterionic coatings) and slippery liquid-infused porous surfaces (SLIPS), for example, create low-energy, hydration-layered interfaces that prevent protein adsorption and bacterial anchorage, thereby arresting colonization at its earliest phase (Table 1) [137]. Bioinspired antimicrobial peptides (AMPs) and their synthetic analogs, covalently grafted to scaffold surfaces, mimic innate immune defenses via selective membrane permeabilization, maintaining persistent bactericidal activity without diffusion into surrounding tissues [138]. Similarly, doped TiO_2_ nanostructures can provide light-triggered ROS generation, thereby enabling spatiotemporally controlled antibacterial activation under physiological or ambient light [139]. Hybrid platforms combining these orthogonal mechanisms—mechanical rupture, ion toxicity, oxidative stress, and fouling resistance—create multifunctional scaffolds that exert robust, synergistic antimicrobial effects with minimal risk of resistance development, marking a substantial advance in infection-resistant regenerative technologies [140].

## 4. Computational Approaches and Future Directions

### 4.1. Multi-Objective Optimization Models

Designing ideal scaffolds for load-bearing tissue interfaces provides a challenging multi-objective optimization problem [104]. Conventional empirical methodologies, such as sequential parametric variations, demonstrate ineffectiveness in investigating the extensive design space created by the numerous factors affecting scaffold performance [28]. Contemporary computational optimization systems address this challenge through advanced algorithms that systematically evaluate millions of potential designs, thereby identifying optimal solutions while considering multiple performance criteria [141]. The transition in scaffold development from intuition-driven to computation-guided design signifies a fundamental shift enabling the construction of buildings with previously unattainable feature combinations [79]. These explicitly acknowledge trade-offs among conflicting needs due to their multi-objective nature, thereby generating Pareto-optimal solution sets that represent the most effective compromises among various objectives [80]. Combining finite element analysis (FEA) with optimization techniques allows exact control of mechanical property distributions across scaffold volumes [81]. By simulating the complicated behavior of porous structures under physiological load circumstances, these computational methods predict stress and strain distributions with great spatial resolution [83]. These models suggest strategic material distribution and structural layouts that enhance load-bearing capacities and minimize stress-shielding effects when coupled with optimization frameworks (Table 2) [84]. Improved material models, including viscoelasticity, anisotropy, and nonlinear behavior unique to both scaffold materials and real tissues [85], can increase the accuracy of these forecasts.

### 4.2. Personalized Scaffold Platforms

Optimizing optimal scaffolds for load-bearing tissue interfaces is a difficult multi-objective challenge [104]. Sequential parametric variations are one of the conventional empirical approaches that show ineffectiveness in exploring the large design space generated by the various aspects influencing scaffold performance [28]. Modern computational optimization systems solve this problem through sophisticated algorithms that methodically assess millions of potential designs [79,141]. Due to their multi-objective character, they openly allow trade-offs between competing demands, thereby producing Pareto-optimal solution sets that best balance among many research goals [80]. Exact control of mechanical property distributions throughout scaffold volumes is potentially possible by combining finite element analysis (FEA) with optimization methods [81]. These computer approaches anticipate stress and strain distributions with remarkable spatial precision by modeling the complex behavior of porous materials under physiological load conditions (Table 2) [83]. Thus, these models provide structural layouts and strategic material distribution that, when combined with optimization frameworks, increase load-bearing capabilities and reduce stress-shielding effects [84]. These predictions may be more accurate using better material models integrating viscoelasticity, anisotropy, and nonlinear behavior special to both scaffold materials and actual tissues [85].

### 4.3. In Silico Mechanobiological Testing

Predictive modeling of biological responses to man-made environments is made possible by digital simulation of cell-scaffold interactions, therefore expediting design iterations and reducing dependence on in vivo experiments [109]. The integration of continuous and discrete mechanobiological models facilitates the capture of multiscale interactions among scaffold design, mechanical stress, and cellular function within these in silico frameworks (Table 2) [142]. Finite element or agent-based models of mechanical–sensory mechanisms, such as integrin-mediated focal adhesion dynamics, cytoskeletal tension transduction, and stretch-activated ion channel activation, predict emergent biological outcomes, including cell migration, differentiation, matrix deposition, and vascular ingrowth [111]. From ossification patterns to fibrous encapsulation risk, scaffold-induced strain energy density, shear stress distributions, and pore-scale fluid dynamics are coupled to biochemical signaling models to estimate spatially and temporally evolving tissue phenotypes (Table 2) [112].

## 5. Discussion and Conclusions

Scaffold technologies for load-bearing tissue interfaces are undergoing a paradigm shift, which is being driven by interdisciplinary developments in materials science, manufacturing, sensor integration, and computational modeling [113,114]. Conventional static scaffolds are being replaced by next-generation platforms that are actively involved in the regeneration process, providing not only mechanical support but also biological guidance, real-time diagnostics, and therapeutic capability [115]. This integrated approach is of paramount importance in challenging clinical scenarios, such as high-load contexts, massive bone defects, and impaired vascularity, where multifunctionality significantly enhances healing outcomes [116]. However, there is still a substantial emphasis on the optimization of mechanical properties, particularly in order to mitigate stress shielding [117]. Mechanical performance and customized biological habitats are both provided by novel scaffold designs that feature hierarchical structures that span macro- to nanoscale dimensions and graded rigidity [118]. Simultaneously, advancements in degradation dynamics have resulted in the development of intelligent materials that can synchronize their degradation with the regrowth of tissue [143]. These adaptive systems actively develop during the healing process by adjusting the degradation rate in accordance with local biological activity, thereby offering patient-specific support [120].

The integration of sensory features into scaffold matrices is similarly transformative [141]. Embedded strain and infection sensors enable the prompt detection of early mechanical failure or bacterial colonization, thereby facilitating therapeutic intervention [122]. These features enhance patient monitoring and offer a previously unheard-of comprehension of in vivo healing processes, thereby informing the development of future scaffolds [123]. Simultaneously, the efficacy of regeneration is enhanced by the use of intricate surface modifications, such as osteoinductive coatings and immunomodulatory and antimicrobial surfaces, which promote positive cellular interactions and, as a result, reduce issues [124].

The precision enabled by additive manufacturing has opened up new design opportunities by enabling the creation of sophisticated, patient-specific shapes and interior structures with spatially controlled material characteristics [125]. Designers can rapidly refine and customize scaffold elements to meet specific clinical needs when combined with computer optimization and machine learning [126]. Regulatory pathways are evolving to accommodate these hybrid, multifunctional products as their therapeutic and financial value becomes more apparent [127].

In all, the combination of these converging technologies signifies a fundamental shift in scaffold design, transitioning from passive constructions to intelligent, therapeutic platforms [128]. As integration across disciplines intensifies, scaffold systems are poised to revolutionize skeletal tissue engineering by providing comprehensive, personalized, and responsive solutions to the most challenging regeneration challenges [144].

## Figures and Tables

**Table 1:** Emerging technologies in smart scaffold platforms: a classification of sensor-integrated and antimicrobial scaffold systems by core technologies, functional capabilities, clinical applications, and innovation trajectories. This table synthesizes recent advances in the development of bioresponsive scaffolds incorporating microsensor networks, biochemical environment tracking, infection detection systems, and antibacterial nanostructures. Each scaffold class is characterized based on its underlying engineering mechanisms, specific sensing or antimicrobial functionalities, clinical use cases, and ongoing innovations driving the field toward autonomous, precision-guided regenerative implants.

Category	Core Technologies	Functional Capabilities	Clinical Applications	Emerging Innovations
**Microsensor Networks for Strain Detection**	Piezoresistive carbon nanocomposites, optical fiber Bragg gratings, graphene sensors, MEMS	Detect micromotion, stress shielding, load transmission; strain <0.1 μm resolution	Monitor spinal fusion, arthroplasty loosening, fracture nonunion strain development	Wireless telemetry, piezoelectric energy harvesting, microbatteries, AI-guided data interpretation
**Biofilm Detection and Infection Control**	EIS, SERS, pH/thermal sensors, quorum sensing probes, machine learning classifiers	Detect early colonization, identify bacterial species, map infection progression	Early diagnosis of implant-associated infections, trigger-localized antimicrobial release	Smart hydrogels, dynamic antimicrobial coatings, multimodal sensing with AI
**pH and Metabolite Tracking**	Fluorophores, ion-selective electrodes, enzymatic glucose/lactate sensors, optical oxygen phosphors	Monitor pH, O_2_, glucose, lactate, cytokines, MMPs; define metabolic/inflammatory profiles	Detect ischemia, inflammation, regeneration quality, scaffold remodeling	Integrated microfluidics, hydrogel multiplex sensing, colorimetric optical diagnostics
**Antibacterial Nanostructures**	ZnO nanowires, nanopillars, silver/copper nanostructures, AMPs, TiO_2_ ROS platforms	Direct bacterial kill via rupture/ROS/ions; prevent adhesion with antifouling coatings	Prevent implant infection without antibiotics, avoid resistance, preserve healing	Hybrid multi-modal platforms, light-triggered antimicrobials, biomimetic surface chemistry

**Table 2:** Taxonomy of computational paradigms in scaffold design: functions, innovations, challenges, and future directions. This table presents a structured classification of leading computational approaches used in next-generation scaffold development. Each paradigm is evaluated across its functional role in scaffold engineering, the novel capabilities it introduces, current technical or translational barriers, and forward-looking research vectors. By organizing these paradigms—ranging from multi-objective optimization to in silico mechanobiological modeling and personalized scaffold generation—this taxonomy highlights the evolving interplay between biomechanics, computation, and precision regenerative design.

Computational Paradigm	Core Functions	Innovations Introduced	Unresolved Challenges	Future Research Vectors
**Multi-Objective Optimization (MOO)**	Design space exploration; performance trade-off balancing	Pareto-optimal scaffold configurations; data-driven optimization replacing trial-and-error	Integration with real-time clinical feedback; interpretability of high-dimensional design spaces	Reinforcement learning-guided optimization; AI-human co-design platforms
**Finite Element Analysis (FEA)-Driven Optimization**	Simulating mechanical behavior under physiological loads	Stress-shielding minimization via spatially distributed material properties	Accurate modeling of anisotropy and viscoelasticity in scaffold-tissue interfaces	Coupling with time-dependent degradation models and real patient load profiles
**In Silico Mechanobiological Simulation**	Predicting biological outcomes (e.g., osteogenesis, vascularization) via mechanical–biochemical coupling	Multiscale modeling of cell–matrix interaction; digital twin of healing environments	Experimental validation of cellular mechanosensitivity at tissue scale	Hybrid models combining agent-based systems with deep mechanotransduction networks
**Personalized Scaffold Modeling**	Subject-specific optimization based on anatomical and loading data	Patient-matched design using computational pipelines from imaging to 3D printing	Scalability of personalization; integration of biological remodeling processes	Closed-loop biofabrication using real-time sensor feedback and AI correction algorithms

## Data Availability

No new data were created or analyzed in this study. Data sharing is not applicable to this article.

## References

[1] Langer R., Vacanti J.P. (1993). Tissue engineering. Science.

[2] Amini A.R., Laurencin C.T., Nukavarapu S.P. (2012). Bone tissue engineering: Recent advances and challenges. Crit. Rev. Biomed. Eng..

[3] O’Brien F.J. (2011). Biomaterials & scaffolds for tissue engineering. Mater. Today.

[4] Hollister S.J. (2005). Porous scaffold design for tissue engineering. Nat. Mater..

[5] Murphy S.V., Atala A. (2014). 3D bioprinting of tissues and organs. Nat. Biotechnol..

[6] Qazi T.H., Mooney D.J., Pumberger M., Geissler S., Duda G.N. (2015). Biomaterials based strategies for skeletal muscle tissue engineering: Existing technologies and future trends. Biomaterials.

[7] Ashammakhi N., Ndreu A., Nikkola L., Wimpenny I., Yang Y. (2008). Advancing tissue engineering by using electrospun nanofibers. Regen. Med..

[8] Place E.S., George J.H., Williams C.K., Stevens M.M. (2009). Synthetic polymer scaffolds for tissue engineering. Chem. Soc. Rev..

[9] Zhang L., Webster T.J. (2009). Nanotechnology and nanomaterials: Promises for improved tissue regeneration. Nano Today.

[10] Bose S., Roy M., Bandyopadhyay A. (2012). Recent advances in bone tissue engineering scaffolds. Trends Biotechnol..

[11] Karageorgiou V., Kaplan D. (2005). Porosity of 3D biomaterial scaffolds and osteogenesis. Biomaterials.

[12] Woodruff M.A., Hutmacher D.W. (2010). The return of a forgotten polymer—Polycaprolactone in the 21st century. Prog. Polym. Sci..

[13] Gentile P., Chiono V., Carmagnola I., Hatton P.V. (2014). An overview of poly(lactic-co-glycolic) acid (PLGA)-based biomaterials for bone tissue engineering. Int. J. Mol. Sci..

[14] Mota C., Puppi D., Chiellini F., Chiellini E. (2015). Additive manufacturing techniques for the production of tissue engineering constructs. J. Tissue Eng. Regen. Med..

[15] Thavornyutikarn B., Chantarapanich N., Sitthiseripratip K., Thouas G.A., Chen Q. (2014). Bone tissue engineering scaffolding: Computer-aided scaffolding techniques. Prog. Biomater..

[16] Lutolf M.P., Hubbell J.A. (2005). Synthetic biomaterials as instructive extracellular microenvironments for morphogenesis in tissue engineering. Nat. Biotechnol..

[17] Engler A.J., Sen S., Sweeney H.L., Discher D.E. (2006). Matrix elasticity directs stem cell lineage specification. Cell.

[18] Frith J.E., Kusuma G.D., Carthew J., Li F., Cloonan N., Gomez G.A., Cooper-White J.J. (2018). Mechanically-sensitive miRNAs bias human mesenchymal stem cell fate via mTOR signalling. Nat. Commun..

[19] Wu R., Li Y., Shen M., Yang X., Zhang L., Ke X., Yang G., Gao C., Gou Z., Xu S. (2021). Bone tissue regeneration: The role of finely tuned pore architecture of bioactive scaffolds before clinical translation. Bioact. Mater..

[20] Moroni L., Burdick J.A., Highley C., Lee S.J., Morimoto Y., Takeuchi S., Yoo J.J. (2018). Biofabrication strategies for 3D in vitro models and regenerative medicine. Nat. Rev. Mater..

[21] Venkatesan J., Anil S., Kim S.K., Shim M.S. (2017). Chitosan as a vehicle for growth factor delivery: Various preparations and their applications in bone tissue regeneration. Int. J. Biol. Macromol..

[22] Midha S., Kim T.B., van den Bergh W., Lee P.D., Jones J.R., Mitchell C.A. (2013). Preconditioned 70S30C bioactive glass foams promote osteogenesis in vivo. Acta Biomater..

[23] Petite H., Viateau V., Bensaid W., Meunier A., de Pollak C., Bourguignon M., Oudina K., Sedel L., Guillemin G. (2000). Tissue-engineered bone regeneration. Nat. Biotechnol..

[24] Green D., Howard D., Yang X., Kelly M., Oreffo R.O. (2003). Natural marine sponge fiber skeleton: A biomimetic scaffold for human osteoprogenitor cell attachment, growth, and differentiation. Tissue Eng..

[25] Maia F.R., Bastos A.R., Oliveira J.M., Correlo V.M., Reis R.L. (2022). Recent approaches towards bone tissue engineering. Bone.

[26] Badylak S.F., Freytes D.O., Gilbert T.W. (2009). Extracellular matrix as a biological scaffold material: Structure and function. Acta Biomater..

[27] Subramanian A., Krishnan U.M., Sethuraman S. (2009). Development of biomaterial scaffold for nerve tissue engineering: Biomaterial mediated neural regeneration. J. Biomed. Sci..

[28] Kelly C.N., Miller A.T., Hollister S.J., Guldberg R.E., Gall K. (2018). Design and structure-function characterization of 3D printed synthetic porous biomaterials for tissue engineering. Adv. Healthc. Mater..

[29] Hutmacher D.W., Sittinger M., Risbud M.V. (2004). Scaffold-based tissue engineering: Rationale for computer-aided design and solid free-form fabrication systems. Trends Biotechnol..

[30] Eltom A., Zhong G., Muhammad A. (2019). Scaffold techniques and designs in tissue engineering functions and purposes: A review. Adv. Mater. Sci. Eng..

[31] Dellavia C., Canciani E., Pellegrini G., Tommasato G., Graziano D., Chiapasco M. (2021). Histological assessment of mandibular bone tissue after guided bone regeneration with customized computer-aided design/computer-assisted manufacture titanium mesh in humans: A cohort study. Clin. Implant. Dent. Relat. Res..

[32] Cheng Q., Trangucci R., Nelson K.N., Fu W., Collender P.A., Head J.R., Hoover C.M., Skaff N.K., Li T., Li X. (2020). In situ bone regeneration of large cranial defects using synthetic ceramic implants with a tailored composition and design. Proc. Natl. Acad. Sci. USA.

[33] Lopes D., Martins-Cruz C., Oliveira M.B., Mano J.F. (2018). Bone physiology as inspiration for tissue regenerative therapies. Biomaterials.

[34] Daculsi G., Fellah B., Miramond T., Durand M. (2013). Osteoconduction, osteogenicity, osteoinduction, what are the fundamental properties for a smart bone substitutes. IRBM.

[35] Li Z., Xie M.B., Li Y., Ma Y., Li J.S., Dai F.Y. (2016). Recent progress in tissue engineering and regenerative medicine. J. Biomater. Tissue Eng..

[36] Salvatore L., Gallo N., Natali M.L., Terzi A., Sannino A., Madaghiele M. (2021). Mimicking the hierarchical organization of natural collagen: Toward the development of ideal scaffolding material for tissue regeneration. Front. Bioeng. Biotechnol..

[37] Siddiqui N., Asawa S., Birru B., Baadhe R., Rao S. (2018). PCL-based composite scaffold matrices for tissue engineering applications. Mol. Biotechnol..

[38] Bello A.B., Kim D., Park H., Lee S.H. (2020). Engineering and functionalization of gelatin biomaterials: From cell culture to medical applications. Tissue Eng. Part B Rev..

[39] Zhang X., Zhang L., Li Y., Hua Y., Li Y., Li W., Li W. (2019). Template-assisted, sol-gel fabrication of biocompatible, hierarchically porous hydroxyapatite scaffolds. Materials.

[40] Wikimedia Commons Contributors File: The Use of Solid Freeform Fabrication in Design of Composite Scaffolds (Image). WIKIMEDIA Commons.

[41] Balint R., Cassidy N.J., Cartmell S.H. (2014). Conductive polymers: Towards a smart biomaterial for tissue engineering. Acta Biomater..

[42] Afewerki S., Sheikhi A., Kannan S., Ahadian S., Khademhosseini A. (2019). Gelatin-polysaccharide composite scaffolds for 3D cell culture and tissue engineering: Towards natural therapeutics. Bioeng. Transl. Med..

[43] Alarcin E., Bal-Ozturk A., Avci H., Ghorbanpoor H., Dogan Guzel F., Akpek A., Yesiltas G., Canak-Ipek T., Avci-Adali M. (2021). Current strategies for the regeneration of skeletal muscle tissue. Int. J. Mol. Sci..

[44] Baniasadi H., Mashayekhan S., Fadaoddini S., Haghirsharifzamini Y. (2016). Design, fabrication and characterization of oxidized alginate-gelatin hydrogels for muscle tissue engineering applications. J. Biomater. Appl..

[45] Serpe F., Casciola C.M., Ruocco G., Cidonio G., Scognamiglio C. (2024). Microfluidic fiber spinning for 3D bioprinting: Harnessing microchannels to build macrotissues. Int. J. Bioprint..

[46] Zhao F., Zhang C., Liu J., Liu L., Cao X., Chen X., Lei B., Shao L. (2020). Periosteum structure/function-mimicking bioactive scaffolds with piezoelectric/chem/nano signals for critical-sized bone regeneration. Chem. Eng. J..

[47] Puwanun S., Delaine-Smith R.M., Colley H.E., Yates J.M., MacNeil S., Reilly G.C. (2018). A simple rocker-induced mechanical stimulus upregulates mineralization by human osteoprogenitor cells in fibrous scaffolds. J. Tissue Eng. Regen. Med..

[48] Mohammadi S., Cidonio G. (2023). Unravelling hierarchical patterning of biomaterial inks with 3D microfluidic-assisted spinning: A paradigm shift in bioprinting technologies. Front. Biomater. Sci..

[49] Balakrishnan B., Joshi N., Jayakrishnan A., Banerjee R. (2014). Self-crosslinked oxidized alginate/gelatin hydrogel as injectable, adhesive biomimetic scaffolds for cartilage regeneration. Acta Biomater..

[50] Mohanraj B., Duan G., Peredo A., Kim M., Tu F., Lee D., Dodge G.R., Mauck R.L. (2019). Mechanically-activated microcapsules for ‘on-demand’ drug delivery in dynamically loaded musculoskeletal tissues. Adv. Funct. Mater..

[51] Hung B.P., Hutton D.L., Grayson W.L. (2013). Mechanical control of tissue-engineered bone. Stem Cell Res. Ther..

[52] Tuan H.S., Hutmacher D.W. (2005). Application of micro CT and computation modeling in bone tissue engineering. Comput. Aided Des..

[53] Bryksin A.V., Brown A.C., Baksh M.M., Finn M.G., Barker T.H. (2014). Learning from nature—Novel synthetic biology approaches for biomaterial design. Acta Biomater..

[54] Schlaubitz S., Derkaoui S.M., Marosa L., Miraux S., Renard M., Catros S., Le Visage C., Letourneur D., Amédée J., Fricain J.-C. (2014). Pullulan/dextran/nHA macroporous composite beads for bone repair in a femoral condyle defect in rats. PLoS ONE.

[55] Rezaeeyazdi M., Colombani T., Memic A., Bencherif S.A. (2018). Injectable hyaluronic acid-co-gelatin cryogels for tissue-engineering applications. Materials.

[56] Jahed E., Khaledabad M.A., Almasi H., Hasanzadeh R. (2017). Physicochemical properties of Carum copticum essential oil loaded chitosan films containing organic nanoreinforcements. Carbohydr Polym..

[57] Acevedo C.A., Sánchez E., Orellana N., Morales P., Olguín Y., Brown D.I., Enrione J. (2019). Re-epithelialization appraisal of skin wound in a porcine model using a salmon-gelatin based biomaterial as wound dressing. Pharmaceutics.

[58] Mulbauer G.D., Matthew H.W.T. (2019). Biomimetic scaffolds in skeletal muscle regeneration. Discoveries.

[59] Wikimedia Commons Techniques for 3D Cell Cultures (SVG Image). https://commons.wikimedia.org/w/index.php?title=File:Techniques_for_3D_Cell_Cultures.svg&oldid=491216417.

[60] Ricci C., Mota C., Moscato S., D’Alessandro D., Ugel S., Sartoris S., Bronte V., Boggi U., Campani D., Funel N. (2014). Interfacing polymeric scaffolds with primary pancreatic ductal adenocarcinoma cells to develop 3D cancer models. Biomatter.

[61] Aviss K.J., Gough J.E., Downes S. (2010). Aligned electrospun polymer fibres for skeletal muscle regeneration. Eur. Cell Mater..

[62] Badylak S.F., Dziki J.L., Sicari B.M., Ambrosio F., Boninger M.L. (2016). Mechanisms by which acellular biologic scaffolds promote functional skeletal muscle restoration. Biomaterials.

[63] Bae S.E., Son J.S., Park K., Han D.K. (2009). Fabrication of covered porous PLGA microspheres using hydrogen peroxide for controlled drug delivery and regenerative medicine. J. Control. Release.

[64] Boldrin L., Elvassore N., Malerba A., Flaibani M., Cimetta E., Piccoli M., Baroni M.D., Gazzola M.V., Messina C., Gamba P. (2007). Satellite cells delivered by micro-patterned scaffolds: A new strategy for cell transplantation in muscle diseases. Tissue Eng..

[65] Borselli C., Cezar C.A., Shvartsman D., Vandenburgh H.H., Mooney D.J. (2011). The role of multifunctional delivery scaffold in the ability of cultured myoblasts to promote muscle regeneration. Biomaterials.

[66] Beier J.P., Klumpp D., Rudisile M., Dersch R., Wendorff J.H., Bleiziffer O., Arkudas A., Polykandriotis E., Horch R.E., Kneser U. (2009). Collagen matrices from sponge to nano: New perspectives for tissue engineering of skeletal muscle. BMC Biotechnol..

[67] Jana S., Leung M., Chang J., Zhang M. (2014). Effect of nano- and micro-scale topological features on alignment of muscle cells and commitment of myogenic differentiation. Biofabrication.

[68] Kang H.W., Lee S.J., Ko I.K., Kengla C., Yoo J.J., Atala A. (2016). A 3D bioprinting system to produce human-scale tissue constructs with structural integrity. Nat. Biotechnol..

[69] Carnes M.E., Pins G.D. (2020). Skeletal Muscle Tissue Engineering: Biomaterials-Based Strategies for the Treatment of Volumetric Muscle Loss. Bioengineering.

[70] Eugenis I., Wu D., Rando T.A. (2021). Cells, scaffolds, and bioactive factors: Engineering strategies for improving regeneration following volumetric muscle loss. Biomaterials.

[71] Qazi T.H., Duda G.N., Ort M.J., Perka C., Geissler S., Winkler T. (2019). Cell therapy to improve regeneration of skeletal muscle injuries. J. Cachexia Sarcopenia Muscle.

[72] Barthes J., Özçelik H., Hindié M., Ndreu-Halili A., Hasan A., Vrana N.E. (2014). Cell microenvironment engineering and monitoring for tissue engineering and regenerative medicine: The recent advances. Biomed. Res. Int..

[73] Milner D.J., Cameron J.A. (2013). Muscle repair and regeneration: Stem cells, scaffolds, and the contributions of skeletal muscle to amphibian limb regeneration. Curr. Top. Microbiol. Immunol..

[74] Haase M., Comlekoglu T., Petrucciani A., Peirce S.M., Blemker S.S. (2024). Agent-based model demonstrates the impact of nonlinear, complex interactions between cytokines on muscle regeneration. eLife.

[75] Anderson J.E. (2022). Key concepts in muscle regeneration: Muscle “cellular ecology” integrates a gestalt of cellular cross-talk, motility, and activity to remodel structure and restore function. Eur. J. Appl. Physiol..

[76] Zhao C., Medeiros T.X., Sové R.J., Annex B.H., Popel A.S. (2021). A data-driven computational model enables integrative and mechanistic characterization of dynamic macrophage polarization. iScience.

[77] Rikard S.M., Athey T.L., Nelson A.R., Christiansen S.L.M., Lee J.J., Holmes J.W., Peirce S.M., Saucerman J.J. (2019). Multiscale coupling of an agent-based model of tissue fibrosis and a logic-based model of intracellular signaling. Front. Physiol..

[78] Hannan R.T., Peirce S.M., Barker T.H. (2018). Fibroblasts: Diverse cells critical to biomaterials integration. ACS Biomater. Sci. Eng..

[79] Bakhshandeh B., Sorboni S.G., Ranjbar N., Deyhimfar R., Abtahi M.S., Izady M., Kazemi N., Noori A., Pennisi C.P. (2023). Mechanotransduction in tissue engineering: Insights into the interaction of stem cells with biomechanical cues. Exp. Cell Res..

[80] Zonderland J., Moroni L. (2021). Steering cell behavior through mechanobiology in 3D: A regenerative medicine perspective. Biomaterials.

[81] Li J., Liu Y., Zhang Y., Yao B., Enhejirigala, Li Z., Song W., Wang Y., Duan X., Yuan X. (2021). Biophysical and biochemical cues of biomaterials guide mesenchymal stem cell behaviors. Front. Cell Dev. Biol..

[82] Vijayavenkataraman S., Shuo Z., Fuh J.Y.H., Lu W.F. (2017). Design of three-dimensional scaffolds with tunable matrix stiffness for directing stem cell lineage specification: An in silico study. Bioengineering.

[83] Altmann B., Steinberg T., Giselbrecht S., Gottwald E., Tomakidi P., Bächle-Haas M., Kohal R.-J. (2011). Promotion of osteoblast differentiation in 3D biomaterial micro-chip arrays comprising fibronectin-coated poly(methyl methacrylate) polycarbonate. Biomaterials.

[84] Amnon B., Rajagopal K., Brown A.E., Discher D.E. (2010). How deeply cells feel: Methods for thin gels. J. Phys. Condens. Matter.

[85] Angele P., Yoo J.U., Smith C., Mansour J., Jepsen K.J., Nerlich M., Johnstone B. (2003). Cyclic hydrostatic pressure enhances the chondrogenic phenotype of human mesenchymal progenitor cells differentiated in vitro. J. Orthop. Res..

[86] Aragona M., Panciera T., Manfrin A., Giulitti S., Michielin F., Elvassore N., Dupont S., Piccolo S. (2013). A mechanical checkpoint controls multicellular growth through YAP/TAZ regulation by actin-processing factors. Cell.

[87] Wu W., Zhao Z., Wang Y., Liu M., Zhu G., Li L. (2024). Mechanism research of elastic fixation promoting fracture healing based on proteomics and fracture microenvironment. Bone Jt. Res..

[88] Deng Q., Wu D., Li M., Dong W. (2022). Polysaccharides, as biological macromolecule-based scaffolding biomaterials in cornea tissue engineering: A review. Tissue Cell.

[89] Dhania S., Bernela M., Rani R., Parsad M., Grewal S., Kumari S., Thakur R. (2022). Scaffolds the backbone of tissue engineering: Advancements in use of polyhydroxyalkanoates (PHA). Int. J. Biol. Macromol..

[90] de Kort B.J., Koch S.E., Wissing T.B., Krebber M.M., Bouten C.V.C., Smits A.I.P.M. (2021). Immuno-regenerative biomaterials for in situ cardiovascular tissue engineering—Do patient characteristics warrant precision engineering?. Adv. Drug Deliv. Rev..

[91] Hatton I.A., Galbraith E.D., Merleau N.S.C., Miettinen T.P., Smith B.M., Shander J.A. (2023). The human cell count and size distribution. Proc. Natl. Acad. Sci. USA.

[92] Braun T., Maroli G. (2021). The long and winding road of cardiomyocyte maturation. Cardiovasc. Res..

[93] Kim Y.H., Tabata Y. (2016). Recruitment of mesenchymal stem cells and macrophages by dual release of stromal cell-derived factor-1 and a macrophage recruitment agent enhances wound closure. J. Biomed. Mater. Res. A.

[94] Abolhassani S., Fattahi R., Safshekan F., Saremi J., Hasanzadeh E. (2025). Advances in 4D bioprinting: The next frontier in regenerative medicine and tissue engineering applications. Adv. Healthc. Mater..

[95] Choi H., Choi W.S., Jeong J.O. (2024). A review of advanced hydrogel applications for tissue engineering and drug delivery systems as biomaterials. Gels.

[96] Hammer N., Ondruschka B., Berghold A., Kuenzer T., Pregartner G., Scholze M., Schulze-Tanzil G.G., Zwirner J. (2023). Sample size considerations in soft tissue biomechanics. Acta Biomater..

[97] Keane T.J., Badylak S.F. (2014). Biomaterials for tissue engineering applications. Semin. Pediatr. Surg..

[98] Zwirner J., Ondruschka B., Scholze M., Hammer N. (2020). Mechanical properties of human dura mater in the context of skull biomechanics. J. Mech. Behav. Biomed. Mater..

[99] Sahakyants T., Vacanti J.P. (2020). Tissue engineering: From bench to bedside via commercialization. Front. Bioeng. Biotechnol..

[100] Žiaran S., Danišovič Ľ., Hammer N. (2025). Editorial: Tissue engineering and regenerative medicine: Advances, controversies, and future directions. Front. Bioeng. Biotechnol..

[101] Kozan N.G., Joshi M., Sicherer S.T., Grasman J.M. (2023). Porous biomaterial scaffolds for skeletal muscle tissue engineering. Front Bioeng. Biotechnol..

[102] Tacchi F., Orozco-Aguilar J., Gutiérrez D., Simon F., Salazar J., Vilos C., Cabello-Verrugio C. (2021). Scaffold biomaterials and nano-based therapeutic strategies for skeletal muscle regeneration. Nanomedicine.

[103] Nuge T., Liu Z., Liu X., Ang B.C., Andriyana A., Metselaar H.S.C., Hoque M.E. (2021). Recent advances in scaffolding from natural-based polymers for volumetric muscle injury. Molecules.

[104] Donmazov S., Saruhan E.N., Pekkan K., Piskin S. (2024). Review of machine learning techniques in soft tissue biomechanics and biomaterials. Cardiovasc. Eng. Technol..

[105] Naqvi S.M., McNamara L.M. (2020). Stem cell mechanobiology and the role of biomaterials in governing mechanotransduction and matrix production for tissue regeneration. Front. Bioeng. Biotechnol..

[106] Montoya C., Du Y., Gianforcaro A.L., Orrego S., Yang M., Lelkes P.I. (2021). On the road to smart biomaterials for bone research: Definitions, concepts, advances, and outlook. Bone Res..

[107] Zhang K., Wang S., Zhou C., Cheng L., Gao X., Xie X., Sun J., Wang H., Weir M.D., Reynolds M.A. (2018). Advanced smart biomaterials and constructs for hard tissue engineering and regeneration. Bone Res..

[108] Wang L., Chen D., Jiang K., Shen G. (2017). New insights and perspectives into biological materials for flexible electronics. Chem. Soc. Rev..

[109] Chen Q., Thouas G.A. (2015). Metallic implant biomaterials. Mater. Sci. Eng. R Rep..

[110] Liu X., Zhao K., Gong T., Song J., Bao C., Luo E., Weng J., Zhou S. (2014). Delivery of growth factors using a smart porous nanocomposite scaffold to repair a mandibular bone defect. Biomacromolecules.

[111] Gaharwar A.K., Singh I., Khademhosseini A. (2020). Engineered biomaterials for in situ tissue regeneration. Nat. Rev. Mater..

[112] Zhang Y., Kumar P., Lv S., Xiong D., Zhao H., Cai Z., Zhao X. (2020). Recent advances in 3D bioprinting of vascularized tissues. Mater. Design.

[113] Jin S., Xia X., Huang J., Yuan C., Zuo Y., Li Y., Li J. (2021). Recent advances in PLGA-based biomaterials for bone tissue regeneration. Acta Biomater..

[114] Li Y., Yang C., Zhao H., Qu S., Li X., Li Y.Y. (2014). New developments of Ti-based alloys for biomedical applications. Materials.

[115] Zhang D., Wu X., Chen J., Lin K. (2018). The development of collagen based composite scaffolds for bone regeneration. Bioact. Mater..

[116] Lin K., Zhang D., Macedo M.H., Cui W., Sarmento B., Shen G. (2019). Advanced collagen-based biomaterials for tissue repair and regeneration. Adv. Funct. Mater..

[117] Ma H., Feng C., Chang J., Wu C. (2018). 3D-printed bioceramic scaffolds: From bone tissue engineering to tumor therapy. Acta Biomater..

[118] Yu W., Sun X., Meng H., Sun B., Chen P., Liu X., Zhang K., Yang X., Peng J., Lu S. (2017). 3D printed porous ceramic scaffolds for bone tissue engineering: A review. Biomater. Sci..

[119] Zhang B., Wang L., Song P., Pei X., Sun H., Wu L., Zhou C., Wang K., Fan Y., Zhang X. (2021). 3D printed bone tissue regenerative PLA/HA scaffolds with comprehensive performance optimizations. Mater. Design.

[120] Wang X., Jiang M., Zhou Z., Gou J., Hui D. (2017). 3D printing of polymer matrix composites: A review and prospective. Compos. Part B Eng..

[121] Derakhshanfar S., Mbeleck R., Xu K., Zhang X., Zhong W., Xing M. (2018). 3D bioprinting for biomedical devices and tissue engineering: A review of recent trends and advances. Bioact. Mater..

[122] Lee J.M., Sing S.L., Tan E.Y.S., Yeong W.Y. (2016). Bioprinting in cardiovascular tissue engineering: A review. Int. J. Bioprint..

[123] Zhang Y.S., Arneri A., Bersini S., Shin S.R., Zhu K., Goli-Malekabadi Z., Aleman J., Colosi C., Busignani F., Dell’Erba V. (2016). Bioprinting 3D microfibrous scaffolds for engineering endothelialized myocardium and heart-on-a-chip. Biomaterials.

[124] Duan B., Hockaday L.A., Kang K.H., Butcher J.T. (2013). 3D bioprinting of heterogeneous aortic valve conduits with alginate/gelatin hydrogels. J. Biomed. Mater. Res. A.

[125] Gao Q., He Y., Fu J.Z., Liu A., Ma L. (2015). Coaxial nozzle-assisted 3D bioprinting with built-in microchannels for nutrients delivery. Biomaterials.

[126] Skardal A., Atala A. (2015). Biomaterials for integration with 3-D bioprinting. Ann. Biomed. Eng..

[127] Ahlfeld T., Cidonio G., Kilian D., Duin S., Akkineni A.R., Dawson J.I., Yang S., Lode A., Oreffo R.O.C., Gelinsky M. (2018). Development of a clay-based bioink for 3D cell printing for skeletal application. Biofabrication.

[128] Jakus A.E., Rutz A.L., Jordan S.W., Kannan A., Mitchell S.M., Yun C., Koube K.D., Yoo S.C., Whiteley H.E., Richter C.-P. (2016). Hyperelastic “bone”: A highly versatile, growth factor-free, osteoregenerative, scalable, and surgically friendly biomaterial. Sci. Transl. Med..

[129] Zhang J., Wehrle E., Adamek P., Paul G.R., Qin X.H., Rubert M., Müller R. (2020). Optimization of mechanical stiffness and cell density of 3D bioprinted cell-laden scaffolds improves extracellular matrix mineralization and cellular organization for bone tissue engineering. Acta Biomater..

[130] Kim S.H., Yeon Y.K., Lee J.M., Chao J.R., Lee Y.J., Seo Y.B., Sultan T., Lee O.J., Lee J.S., Yoon S.-I. (2018). Precisely printable and biocompatible silk fibroin bioink for digital light processing 3D printing. Nat. Commun..

[131] Hong H., Seo Y.B., Kim D.Y., Lee J.S., Lee Y.J., Lee H., Ajiteru O., Sultan T., Lee O.J., Kim S.H. (2020). Digital light processing 3D printed silk fibroin hydrogel for cartilage tissue engineering. Biomaterials.

[132] Xue J., Wu T., Dai Y., Xia Y. (2019). Electrospinning and electrospun nanofibers: Methods, materials, and applications. Chem. Rev..

[133] Li X., Wang L., Fan Y., Feng Q., Cui F.Z., Watari F. (2013). Nanostructured scaffolds for bone tissue engineering. J. Biomed. Mater. Res. A.

[134] Hasan A., Waibhaw G., Tiwari S., Dharmalingam K., Shukla I., Pandey L.M. (2017). Fabrication and characterization of chitosan, polyvinylpyrrolidone, and cellulose nanowhiskers nanocomposite films for wound healing drug delivery application. J. Biomed. Mater. Res. A.

[135] Zhang Y., Liu X., Zeng L., Zhang J., Zuo J., Ding J., Chen X. (2019). Polymer fiber scaffolds for bone and cartilage tissue engineering. Adv. Funct. Mater..

[136] Qu J., Zhao X., Liang Y., Zhang T., Ma P.X., Guo B. (2018). Antibacterial adhesive injectable hydrogels with rapid self-healing, extensibility and compressibility as wound dressing for joints skin wound healing. Biomaterials.

[137] Wei Q., Becherer T., Angioletti-Uberti S., Dzubiella J., Wischke C., Neffe A.T., Lendlein A., Ballauff M., Haag R. (2014). Protein interactions with polymer coatings and biomaterials. Angew. Chem. Int. Ed..

[138] Ng W.L., Chua C.K., Shen Y.F. (2019). Print me an organ! Why we are not there yet. Prog. Polym. Sci..

[139] Sun W., Starly B., Daly A.C., Burdick J.A., Groll J., Skeldon G., Shu W., Sakai Y., Shinohara M., Nishikawa M. (2020). The bioprinting roadmap. Biofabrication.

[140] Matai I., Kaur G., Seyedsalehi A., McClinton A., Laurencin C.T. (2020). Progress in 3D bioprinting technology for tissue/organ regenerative engineering. Biomaterials.

[141] Sporn K., Kumar R., Paladugu P., Ong J., Sekhar T., Vaja S., Hage T., Waisberg E., Gowda C., Jagadeesan R. (2025). Artificial Intelligence in Orthopedic Medical Education: A Comprehensive Review of Emerging Technologies and Their Applications. Int. Med. Educ..

[142] Kumar R., Waisberg E., Ong J., Paladugu P., Amiri D., Saintyl J., Yelamanchi J., Nahouraii R., Jagadeesan R., Tavakkoli A. (2024). Artificial Intelligence-Based Methodologies for Early Diagnostic Precision and Personalized Therapeutic Strategies in Neuro-Ophthalmic and Neurodegenerative Pathologies. Brain Sci..

[143] Wang J., Kong X., Li Q., Li C., Yu H., Ning G., Xiang Z., Liu Y., Feng S. (2021). The spatial arrangement of cells in a 3D-printed biomimetic spinal cord promotes directional differentiation and repairs the motor function after spinal cord injury. Biofabrication.

[144] Kumar R., Sporn K., Khanna A., Paladugu P., Gowda C., Ngo A., Jagadeesan R., Zaman N., Tavakkoli A. (2025). Integrating Radiogenomics and Machine Learning in Musculoskeletal Oncology Care. Diagnostics.

